# Supplementary material for: Empirical Model of Spring 2020 Decrease in Daily Confirmed COVID-19 Cases in King County, Washington
Source: medRxiv. 2022 Feb 7:2020.05.11.20098798. Preprint. [Version 4] doi: 10.1101/2020.05.11.20098798 (PMC9216716; doi:10.1101/2020.05.11.20098798)

medRxiv preprint doi: https://doi.org/10.1101/2020.05.11.20098798; this version posted February 7, 2022. The copyright holder for this preprint (which was not certified by peer review) is the author/funder, who has granted medRxiv a license to display the preprint in perpetuity.
It is made available under a CC-BY 4.0 International license .

METHODS AND SUPPLEMENTARY NOTES

Code. All analyses were performed with R version 4.0.1 (R Foundation for Statistical Computing, Vienna, Austria). R code is provided as a supplemental file.

Data. Data is provided as a supplemental file. Data was acquired by manual transcription of the daily confirmed case counts daily from February 28 through June 17, 2020; more automated download was not available (PHSKC website, 2020).

One approach to best capturing the information in these variables is to use the most up-to-date, latest data. Although such data may be more incomplete and less reliable than an earlier data freeze, it may lead to a more useful model by better capturing the latest effects of dynamic changes in hidden variables. Over time, Public Health — Seattle & King County (PHSKC) improves previously reported confirmed case counts by removing duplicates, correcting residency information, adding newly received counts from previous dates, improving cause of death information, and other data cleaning. This creates a potential trade-off between using data that includes the last few reported days and using only older more reliable data. However, for a two-parameter model with many data points, inclusion or exclusion of the last two days of data has little effect on the model. Therefore, the model fit presented here includes all data, to best capture recent changes in underlying parameters, such as changes in population behavior or resolving flares in relatively isolated subpopulations, but at the risk of being more sensitive to issues associated with delayed case reporting and database maintenance.

To date, revisions to the PHSKC daily confirmed case reports fall into three categories: (1) additions to the last several days as new cases arrive in the database, (2) minor revisions to cases counts up to several weeks old, and (3) major revisions to the latest ten days of data. Revisions of the first type tend to have only a minor impact on estimates because they typically only have a noticeable magnitude of change for the last datapoint. They can also be adjusted statistically to add the expected proportion of delayed cases based on historically reported second-day adjustments. Revisions of the second type have negligible impact on estimates, as they seldom alter the counts for a given day by more than one or two counts. The third type of revision has happened only once, over the weekend, between May 2 and May 3, 2020; on May 3 a total of 105 cases counts were subtracted from the previous ten days, biased towards more recent dates, with over 20 counts being subtracted from previously reported counts for April 28, April 29, and April 30. This revision would have substantially altered the conclusions of the approach presented here and represent a major caveat to the interpretation and use of these results; these results will be substantially misleading if major anomalies occur again.

As this model intends to capture early indicators of outbreak progression, it focuses on reported cases counts, not deaths or hospitalizations. Deaths and hospitalizations, although more reliably assayed and reported and so are used in models such as that of the IHME (IHME COVID-19 Health Service Utilization Forecasting Team, 2020), are lagging indicators compared to incident case reports, but are excellent for predicting peak usage of resources.

The model presented here uses data from the PHSKC website. The IDM model uses data obtained directly from WA state. Although the PHSKC website states that it is an exact reflection of state data, there are differences between these data sets. The differences are minor and do not affect major conclusions. The reasons for these differences are not known.

Starting Date. This model is robust to choice of initial data date. For example, for an analysis performed on May 11, choices of start date from March 26 to March 31 all result in a half-life between 24 and 25 days. Choices of later start dates produce longer half-lives, suggesting that the rate of decline may be slowing, however, uncertainty in these estimates rises as they are derived from fewer data points. For example, as of May 11, the half-life estimated from April 10 data and on was 32 days. This also suggests a slowing of the rate of decline during the month of April and early May. The choice of starting date for data fit can be considered a parameter, so it is reasonable to consider the exponential model a three-parameter model – but if that choice is made for comparative purposes then other models should also have data-selection parameters enumerated. If the exponential model were applied to historical datasets, then choice of the end date for the modeled interval would constitute a fourth parameter. Extreme changes in initial date can change model predictions (e.g., **Table 1**, bottom two rows), and suggest a change in R across different time intervals.

Model Fit. The absolute residuals of the model are fairly constant and show little structure. This suggests that data deviating from the model fit is well described as "noise," rather than some systematic effect, such as a change in public policy or public response to that policy (**Figure S1**). However, the residual on May 30 (index #66) was

medRxiv preprint doi: https://doi.org/10.1101/2020.05.11.20098798; this version posted February 7, 2022. The copyright holder for this preprint (which was not certified by peer review) is the author/funder, who has granted medRxiv a license to display the preprint in perpetuity.
It is made available under a CC-BY 4.0 International license .

one of the four highest residuals; it occurred seven days after Memorial Day and could represent a response to social gathering on Memorial Day. However, that seems unlikely because the flanking days do not have similarly high residuals. There is also a mild weekly periodicity in the data, presumably corresponding to testing cycles (**Figure S2**). Therefore, smoothing over at least a week's time is recommended; use of a two-parameter model achieves that goal.

Utility of R as a Public Health Policy Tool When R Hovers Near Unity. The half-life statistic is particularly useful when used separately for pre- and post-peak modeling. R is a more versatile and general statistic that has value in informing policy throughout an outbreak. It is particularly useful if the value of R hovers near 1, as there are profound implications for policy depending upon which side of unity the statistic lies. If R is less than but near 1, half-life is near infinity and is inelegant as a reportable metric. Half-life is much more useful if R is consistently somewhat less than 1, and in these circumstances is a key statistic for planning for healthcare demand and tempo of economic and social adjustments. Similarly, if R is greater than 1, the doubling time statistic grows in value for public communication. Also, due to the nonlinearity of the models, the confidence intervals for R can be asymmetric around the point estimate, increasing the likelihood of misinterpretations across a chain of public communication.

Rigor Metrics for Natural Language Processing. Sex as a biological variable data was not provided by Seattle & King County (PHSKC). Breakdown of daily data into subcategories including sex and ethnicity was not available in part due to privacy concerns related to small aggregate bin sizes (personal communication from PHSKC, May 28, 2020). This study did not require institutional review board review. This human-subjects exempt study did not require consent, randomization, or blinding. A power analysis was not applicable. No cell lines were used; no authentication was necessary.

Key Resources Table

Software and Algorithms

Statement: All analyses were performed with R version 4.0.1 (2020-06-06) (R Foundation for Statistical Computing, Vienna, Austria).

Reagent or Resource: R

Source: NA

Identifier: (R Project for Statistical Computing, RRID:SCR_001905)

medRxiv preprint doi: https://doi.org/10.1101/2020.05.11.20098798; this version posted February 7, 2022. The copyright holder for this preprint
(which was not certified by peer review) is the author/funder, who has granted medRxiv a license to display the preprint in perpetuity.
It is made available under a CC-BY 4.0 International license .

Figure S1. Model residuals. There is little structure to the data, and no significant structure. Therefore deviations of observed real values from model predictions are well described as noise, rather than attributed to failure of modeling assumptions.

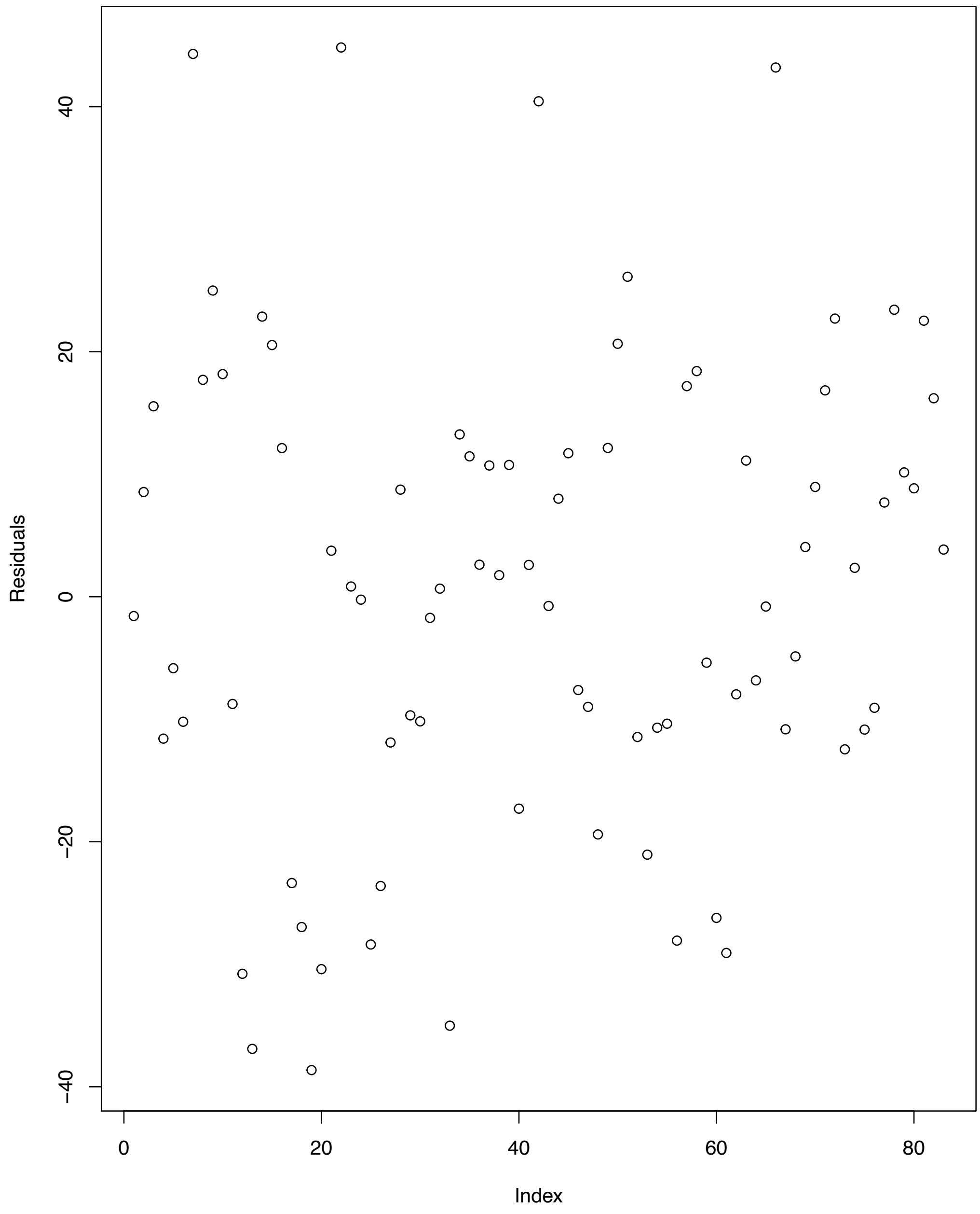

medRxiv preprint doi: https://doi.org/10.1101/2020.05.11.20098798; this version posted February 7, 2022. The copyright holder for this preprint
(which was not certified by peer review) is the author/funder, who has granted medRxiv a license to display the preprint in perpetuity.
It is made available under a CC-BY 4.0 International license .

Figure S2. Autocorrelation of model residuals, as computed by the default parameters of the R `acf` function. Lag is expressed in days. A peak at seven days, and a smaller one at 14 days, indicates that there are weekly testing cycles, such as likely results from changes in availability of testing sites and in personal schedules over the weekend.

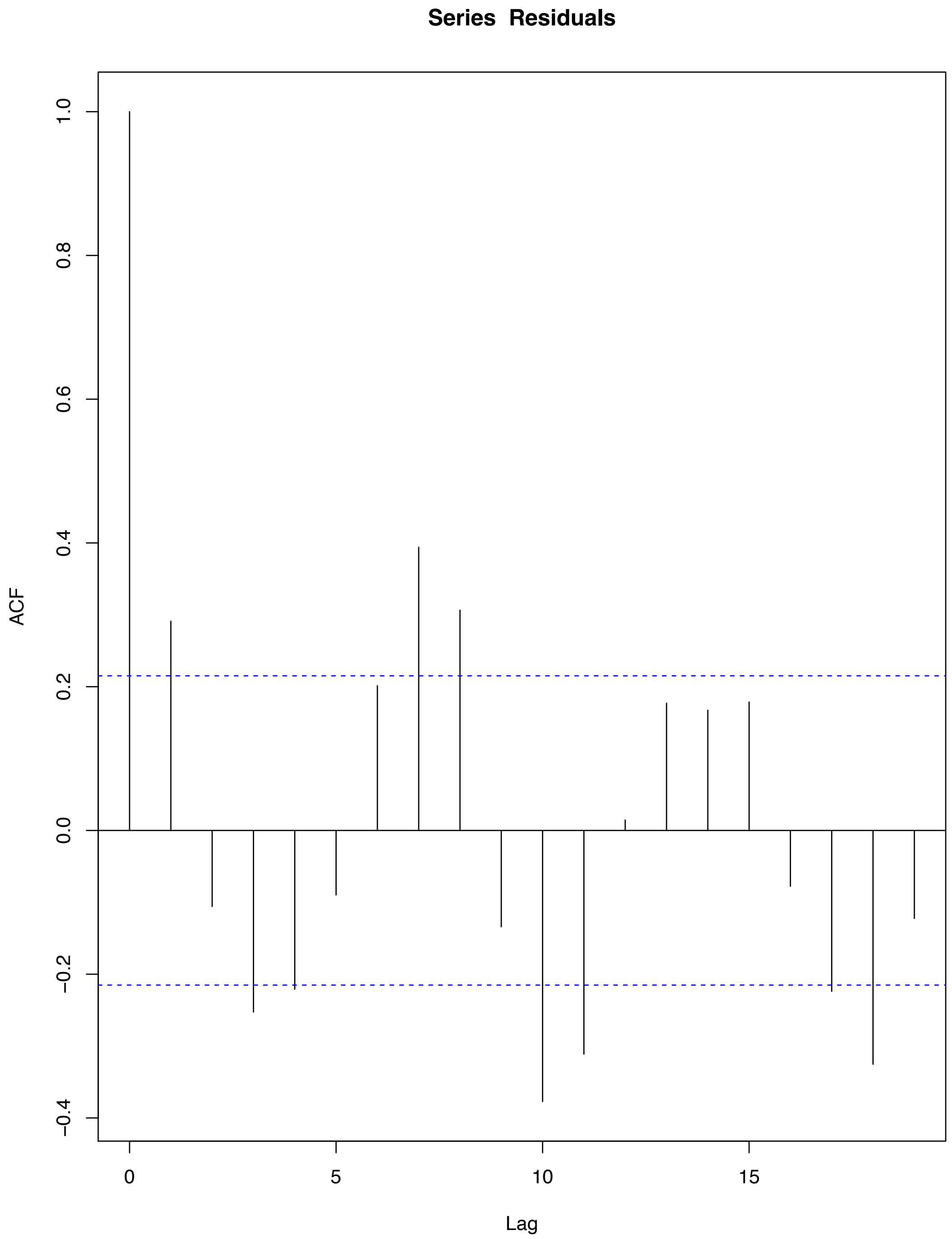

Supplement: 1 [file NIHPP2020.05.11.20098798V4-supplement-1.pdf]
